# Barriers to access and utilization of emergency obstetric care at health facilities in sub-Saharan Africa: a systematic review of literature

**DOI:** 10.1186/s13643-018-0842-2

**Published:** 2018-11-13

**Authors:** Ayele Geleto, Catherine Chojenta, Abdulbasit Musa, Deborah Loxton

**Affiliations:** 1 School of Public Health, College of Health and Medical Sciences, Haramaya University, Harar, Ethiopia; 2 Research Centre for Generational Health and Ageing, School of Medicine and Public Health, Faculty of Health and Medicine, The University of Newcastle, Newcastle, Australia; 3 School of Nursing and Midwifery, College of Health and Medical Sciences, Haramaya University, Harar, Ethiopia

**Keywords:** Barrier, Access, Utilization, Emergency obstetric care, Sub-Saharan Africa

## Abstract

**Background:**

Nearly 15% of pregnancies end in fatal perinatal obstetric complications including bleeding, infections, hypertension, obstructed labour and complications of abortion. Globally, an estimated 10.7 million women have died due to obstetric complications in the last two decades, and two thirds of these deaths occurred in sub-Saharan Africa. Though the majority of maternal mortalities can be prevented, different factors can hinder women’s access to emergency obstetric services. Therefore, this review is aimed at synthesizing current evidence on barriers to access and utilization of emergency obstetric care in sub-Saharan Africa.

**Methods:**

Articles were searched from MEDLINE, CINAHL, EMBASE, and Maternity and Infant Care databases using predefined search terms and strategies. Articles published in English, between 2010 and 2017, were included. Two reviewers (AG and AM) independently screened the articles, and data extraction was conducted using the Joanna Briggs Institute data extraction format. The quality of the included studies was assessed using the Mixed Methods Appraisal Tool. The identified barriers were qualitatively synthesized and reported using the Three Delays analytical framework. The PRISMA checklist was employed to present the findings.

**Result:**

The search of the selected databases returned 3534 articles. After duplicates were removed and further screening undertaken, 37 studies fulfilled the inclusion criteria. The identified key barriers related to the first delay included younger age, illiteracy, lower income, unemployment, poor health service utilization, a lower level of assertiveness among women, poor knowledge about obstetric danger signs, and cultural beliefs. Poorly designed roads, lack of vehicles, transportation costs, and distance from facilities led to the second delay. Barriers related to the third delay included lack of emergency obstetric care services and supplies, shortage of trained staff, poor management of emergency obstetric care provision, cost of services, long waiting times, poor referral practices, and poor coordination among staff.

**Conclusions:**

A number of factors were found to hamper access to and utilization of emergency obstetric care among women in sub-Saharan Africa. These barriers are inter-dependent and occurred at multiple levels either at home, on the way to health facilities, or at the facilities. Therefore, country-specific holistic strategies including improvements to healthcare systems and the socio-economic status of women need to be strengthened. Further research should focus on the assessment of the third delay, as little is known about facility-readiness.

**Systematic review registration:**

PROSPERO CRD42017074102

**Electronic supplementary material:**

The online version of this article (10.1186/s13643-018-0842-2) contains supplementary material, which is available to authorized users.

## Background

Existing evidence indicated that 15% of pregnant women develop some form of obstetric complications during pregnancy and childbirth which is likely to result in maternal death if they fail to receive rapid obstetric interventions [1]. The majority of maternal mortalities are caused by direct obstetric events such as haemorrhage, hypertension, obstructed labour, sepsis, and complications of abortion [2]. The World Health Organization’s (WHO) report indicated that globally in 2015, an estimated 303,000 women died due to obstetric complications [3]. While almost all of the global maternal deaths occurred in developing countries, about two thirds of these deaths took place in sub-Saharan Africa [4].

Though the occurrence of obstetric complications is often unpredictable [5], there is evidence that maternal mortality can be prevented [6]. Prevention of maternal mortality can be realized through making pregnancy and childbirth safer by ensuring that women who face obstetric complications have access to timely obstetric care [7]. Emergency obstetric care (EmOC) is an evidence-based service required to manage potentially life-threatening complications that affect many women during pregnancy, childbirth, and the immediate postpartum period [8, 9].

There are two complementary types of EmOC facilities: basic emergency obstetric care (BEmOC) and comprehensive emergency obstetric care (CEmOC) facilities [9]. A BEmOC facility can provide six crucial obstetric services, known as “signal functions”, which include administration of parenteral antibiotics, parenteral anticonvulsants, and parenteral uterotonics; removal of retained products; manual removal of the placenta; and assisted vaginal delivery (AVD). A CEmOC facility provides caesarean sections and blood transfusions, in addition to the six signal functions of BEmOC [8].

It has been suggested that the execution of EmOC has resulted in a noticeable global reduction of the maternal mortality ratio (MMR) since 1990 [10]. However, there was a significant regional variation in MMR across the globe, where sub-Saharan Africa still sustains a large MMR [3]. Recognizing this problem, the United Nations (UN) has set a new global strategy, the Sustainable Development Goals (SDGs), which are aimed at reducing the global MMR to fewer than 70 per 100,000 live births by 2030 [11]. This target will not be achieved unless the provision of quality EmOC is strengthened, especially in sub-Saharan Africa where the MMR is currently 546 per 100,000 live births [3].

Nevertheless, findings of research in developing countries have indicated poor utilization of EmOC among women who have experienced obstetric complications. In a study conducted in nine sub-Saharan African countries, researchers showed that only 28% of women who experienced obstetric complications obtained EmOC [12]. In a recent study in Tanzania, it was indicated that the met need for emergency obstetric care was 22% [13] while the met need of EmOC for Malawian and Zambian women was reported to be 20.7% [14] and 27% [15] respectively. In Ethiopia, the findings of Admasu et al. (2011) showed that only 6% of women who experienced obstetric complications were treated at health institutes [16].

In sub-Saharan Africa, access to and utilization of EmOC services among women are affected by several factors. Unavailability of the services is the most important factor that influenced utilization of services among women [15–17]. Other barriers to access and utilization of EmOC services included a woman’s lack of knowledge about pregnancy complications [18, 19] and women’s poor awareness of the availability of EmOC services [20, 21]. Poor quality of EmOC services, as defined by a lack of essential medical supplies [22] and lack of training among healthcare providers resulting in a lack of competency [23], was frequently reported to affect utilization of EmOC services. In the majority of studies conducted in sub-Saharan Africa, researchers concluded that the quality of EmOC services is usually poor, as indicated by higher direct obstetric case fatality rates than the UN standard of not more than 1% [23–25].

**Fig. 1:**
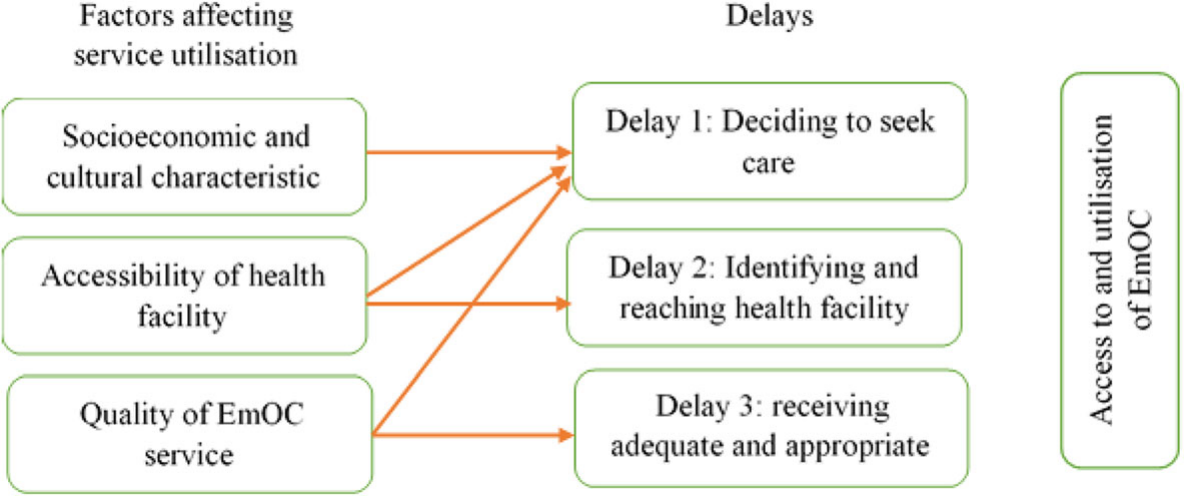
The three delays model

The three delays model is an ideal framework for conceptualizing the various barriers to access and utilization of EmOC services (Fig. 1). This model can capture factors that cause delays in seeking obstetric care and has been employed in several studies [26, 27]. In this model, three types of delays are proposed: the delays that could occur either at home, on the way to the health facility, or in the health facility [28]. Socio-cultural factors, accessibility and affordability of the service, and quality of care may independently affect the lengths of each delay.

Limited previous reviews exist on obstetric health services in sub-Saharan Africa, and no reviews to date have specifically focused on barriers to access and utilization of EmOC services. While some reviews have focused on accessing barriers to obstetric care [29], these have not specifically been in relation to EmOC services. Rather, researchers have focused on facilitators and barriers of facility delivery [30] and the application of international guidelines for EmOC [31]. Therefore, this systematic review was aimed at identifying and presenting factors affecting access to and utilization of EmOC services at health facilities in sub-Saharan Africa.

## Methods

The study protocol of this review was registered in PROSPERO 2017 and is available at http://www.crd.york.ac.uk/PROSPERO/display_record.asp?ID=CRD42017074102, ID = CRD42017074102. The review protocol was also published on BMC systematic review and can be found at https://systematicreviewsjournal.biomedcentral.com/track/pdf/10.1186/s13643-018-0720-y. The Preferred Reporting Items for Systematic Reviews and Meta-Analyses (PRISMA) checklist [32] was employed to present the findings of studies on barriers to utilization of EmOC in sub-Saharan Africa.

### Data sources and search strategy

A systematic search was conducted to obtain appropriate published articles about barriers to access and utilization of EmOC in sub-Saharan Africa. Electronic databases including MEDLINE, CINAHL, EMBASE, and Maternity and Infant Care were searched to identify appropriate articles. In consultation with the faculty librarian (DB), we retrieved articles published in the English language between January 2010 and August 2017 using appropriate Boolean operators. Searching for articles was conducted in August 2017, and the MEDLINE database search strategy and terms are presented in Additional file 1. This systematic review used the Population, Intervention, Comparison, Outcomes and Study (PICOS) setting framework to determine the eligibility of the articles. Participant (P) refers to mothers who experienced obstetric complications and who did not access EmOC services, while the Intervention (I) was EmOC. The Comparison (C) was those mothers who experienced obstetric complications and received EmOC services, the Outcome (O) was barriers to access and utilization of EmOC services, and the study Setting (S) was sub-Saharan Africa.

The following key terms were used in combination or separately to find suitable articles from different electronic databases: “Emergency Obstetric Care” OR “Emergency Obstetric and Newborn Care” OR EmOC OR EmONC OR “pregnancy complication*” OR “obstetric complication*” OR “maternal ha?morrhage” OR “pregnancy induced hypertension” Eclampsia OR Pre-eclampsia OR “maternal infection” OR “obstructed labo?r” OR “complication* of abortion” OR “cesarean section” OR “manual vacuum extraction” OR Oxytocin OR “magnesium sulphate” AND (barrier* OR obstacle* OR factor* OR Challenge* OR determinant* OR access* OR utiliz* OR Utilis* OR hinder* OR hindrance* OR impede* OR impediment*).mp. AND “sub-Saharan Africa” OR “Africa South of Sahara” to locate relevant articles for this systematic review. The reference lists of eligible papers were also searched manually and were included in the review.

### Eligibility criteria

All articles which reported on barriers to access and utilization of EmOC services from a service users’ perspective, and challenges to providing EmOC services at health facilities, were included. In other words, all articles that reported any factors that delayed mothers at home, on the way to the health facility, and at the facility from receiving timely emergency obstetric service were included in the review. We included papers published between January 2010 and August 2017 in order to capture barriers to access and utilization of EmOC after the update of the WHO handbook for “Monitoring Emergency Obstetric Care”, which was released in 2009 [9]. Both quantitative and qualitative studies that were conducted with a cross-sectional study design in sub-Saharan Africa were included, irrespective of whether the study was conducted in a health facility or in the community.

### Exclusion criteria

Articles published in languages other than English and where the data collection period took place before January 2010, or in a country outside of sub-Saharan Africa, were excluded. Studies that reported barriers to the utilization of obstetric care that have women with no obstetric complications as the population of interest were similarly excluded. Commentaries and anonymous reports were also excluded from this systematic review.

### Screening of the articles

Results from the initial searches were stored in an EndNote library. After removing duplicated articles, the EndNote library was shared between the two reviewers (AG and AM) to independently screen the articles by title and abstract, guided by the eligibility criteria. Those studies that the two reviewers agreed on were included in the full-text review. Disagreement between the two reviewers was handled by discussion. The two reviewers independently reviewed the full text of the eligible papers. Finally, the full texts of all relevant articles that were found to meet the inclusion criteria were retained for the narrative synthesis.

### Quality appraisal

The Mixed Methods Appraisal Tool (MMAT) [33] was used to assess the quality of the identified papers. This quality assessment tool was found to have a moderate to perfect inter-rater reliability score [34] and has been used in different systematic reviews of mixed-method design [29, 35]. The MMAT was designed to assess the quality of articles that were conducted with qualitative, quantitative, and mixed-methods designs. There are four quality assessment criteria for qualitative and quantitative studies, and the quality of each study is determined by dividing the number of criteria met by four. For mixed-methods studies, the premise is that the overall quality of a combination cannot exceed the quality of its weakest component. Thus, the overall quality score for mixed-methods designs is the lowest score of the study components [33]. Quality scores for each article are presented in [Additional file 2].

### Data extraction

Data were extracted from the full text of retained articles using an adapted Joanna Briggs Institute (JBI) data extraction form [36]. Study characteristics including the name of the first author and publication year, data collection period, and the country in which the study conducted were extracted. Specific study details such as study design, study population, sample size, sampling procedure, data collection procedure, and response rate were captured. The detail of factors reported as barriers to access and utilization of EmOC services were exhaustively extracted from the included articles [Additional file 3].

### Data synthesis

A narrative synthesis approach [37] was employed to conduct and present the findings of this systematic review. The three delays model was used as the framework in order to identify the barriers to access and utilization of EmOC services [28]. Finally, summary tables were generated from crude data on barriers to access and utilization of EmOC services (Table 1). The identified barriers were coded to illustrate the relationship between a substantive factor and the specific delays. Origin words from the extracted data were used to label the codes. Thereafter, codes with similar meanings were clustered together into categories. Finally, the theoretical model that explained the phenomenon under study (the three delay model) were identified as themes, and qualitative case descriptions were performed. All steps in article identification and report writing followed the PRISMA statement [32].

**Table 1:** Thematic summary of barriers to access and utilization of EmOC in different sub-Saharan Africa

Main themes	The emerged sub-themes	Factors contributing to the delay in seeking EmOC services	Studies
Delay I	Socio-demographic and economic factors	Young age	[53, 59]
		Uneducated women	[49, 73]
		Attending only primary/secondary education	[53, 61, 62]
		Unemployment	[53, 61, 62]
		Rural residence	[40, 47, 49, 53, 64, 66, 67, 69]
		Poverty and low income	[39, 40, 49, 51, 59, 63]
		Unmarried	[53]
		Language issues	[49]
		Lack of information about service	[49, 51]
		Being occupied with harvest and other duties	[46]
	Community perception about obstetric complications	Socio-cultural	[48, 49, 63, 65, 72]
		Belief in alternative method	[39, 45, 58, 68, 73]
		Negative perception of the service	[53, 66]
		Social stigma	[48]
		Lack of trust in HCW	[44, 65]
		Expecting improvement over time	[63]
		Fear of procedure like surgery and blood donation	[38, 51, 58, 72]
		Desire for home delivery	[45, 58]
	Lack of women’s autonomy and poor male involvement	Women not involved in decision-making	[46, 49]
		Poor male involvement	[43, 61, 62]
	Knowledge of obstetric danger signs	Lack of awareness about obstetric complications	[39, 45, 49, 57, 59, 63, 66, 68, 72, 73]
		Inability to identify complications	[49, 53, 59, 61, 62, 64, 68, 73]
	Obstetric history and health service use	Inadequate ANC use	[39, 45, 59, 61, 62]
		Higher parity and gravidity	[51, 59, 62, 73]
		Previous uncomplicated pregnancy	[46, 66]
		Poor birth preparedness and complication readiness	[39, 57]
		Unwanted pregnancy	[53]
		Previous bad experiences at facility and dissatisfaction	[51, 60, 72]
Delay II	Poor transport infrastructure	Lack of vehicles	[39, 45, 49, 51, 59, 65, 66, 68]
		Shortage of ambulances	[51, 56, 66, 69, 72]
		Poor road infrastructure and geography	[52, 63, 66, 68, 72]
	Distance from health facilities	Long distance from facility	[42, 46, 51–53, 60, 63, 64, 68]
		Lack of health facility in rural area	[49, 65, 67, 69]
		Poor referral communication	[23, 42, 72]
		Sought care first from dispensary or health centre	[51, 54]
	Lack of finance for transportation	Lack of money for transportation	[42, 45, 46, 51, 58, 63, 66, 70, 72]
Delay III	Lack of EmOC services and supplies	Unavailability of EmOC services	[41, 47, 48, 51, 65, 67, 69, 70]
		Lack of drugs, medical supplies, and equipment	[41, 47–50, 55, 56, 60, 68–71]
		Shortage of rooms and utilities	[38, 41, 44, 46, 49–51, 60, 71]
		Lack of blood	[41, 45, 63, 68, 69]
		Sub-standard care at facility	[39, 45, 58, 65, 68–72]
	Healthcare providers’ training and attitude	Shortage of healthcare providers	[38, 41, 44, 48–51, 54, 56, 60, 65, 69, 71]
		Lack of competence among providers	[23, 44, 45, 50, 56, 60, 72]
		Misdiagnosis and inappropriate treatment	[39, 45, 58, 69, 70, 72]
		Shortage of training	[41, 44, 48, 50, 51, 56, 60, 69–72]
		Long waiting time	[41, 45, 52, 65, 66, 68]
		Provider’s poor attitude	[38, 44, 46, 48, 51, 54, 55, 58, 60, 65]
		Lack of privacy	[41, 44, 58, 60, 65]
	Poor management system	Poor supportive supervision	[23, 48, 50, 69]
		Poor staff motivation	[41, 51, 54, 55, 69, 72]
		Staff absenteeism	[41, 54]
		Lack of coordination and feedback	[41, 51, 54, 55, 69, 72]
		Heavy workload	[48, 49, 54, 69]
		High staff turnover	[23, 69]
		Poor communication system	[44, 60, 65, 72]
		Patient overcrowding	[23, 38, 41, 46]
		Delayed referral	[41, 42, 45, 68, 69]
		Lack of guidelines and protocol	[48, 50, 54, 71]
		Unaccountability	[54, 55, 69]
		High treatment cost	[38, 39, 58, 60, 72]
		Simplicity of obtaining drugs	[60]

## Results

**Fig. 2:**
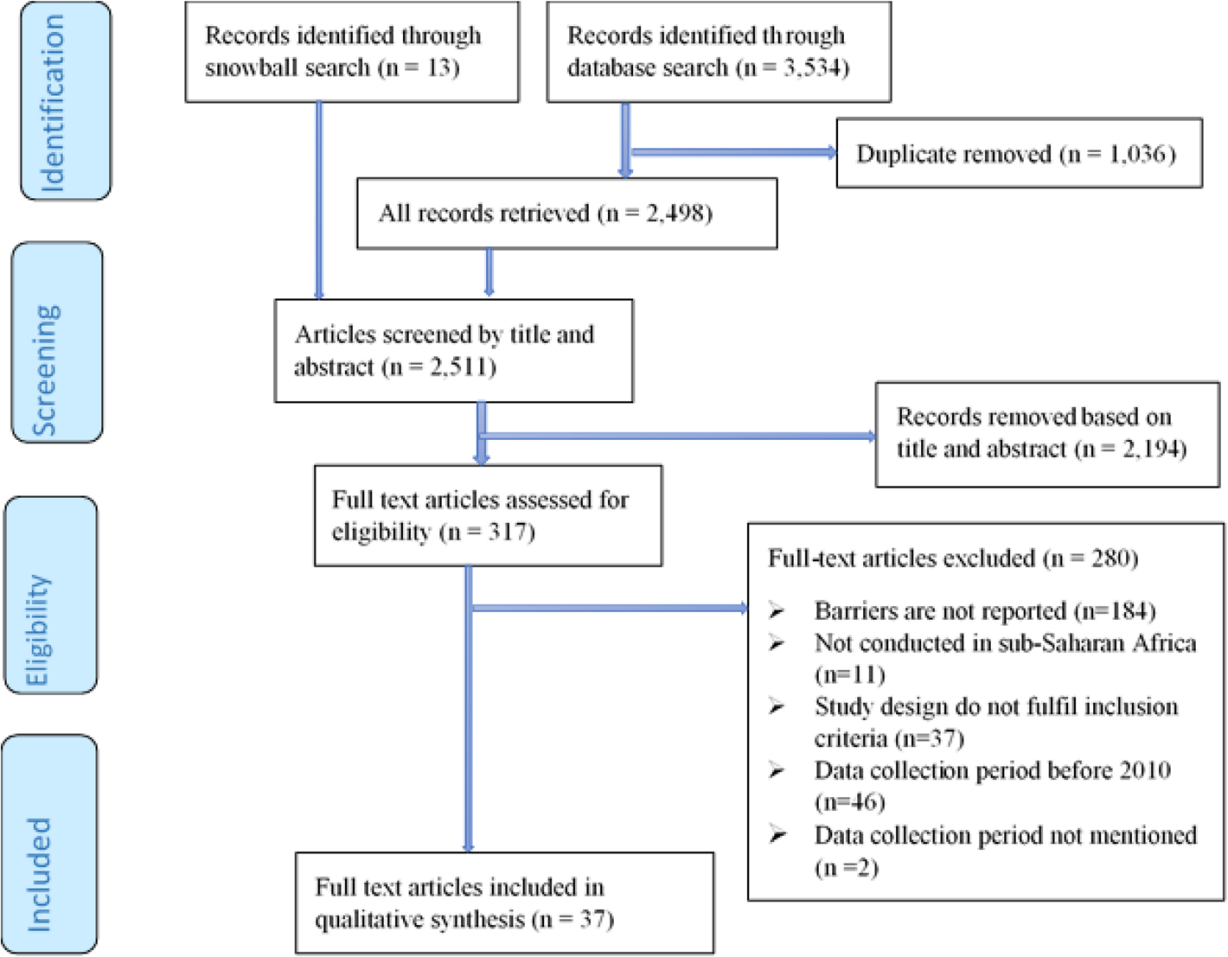
PRISMA flow diagram indicating screening of the articles

The search of the selected databases initially returned 3534 articles. After 1036 duplicates were removed, 2498 articles remained for further screening and 13 additional studies were located through checking the reference lists of the identified papers. Finally, 317 articles were retrieved for full-text review, of which 280 studies were excluded for not meeting the eligibility criteria. Thirty-seven studies [38–73] were retained for the final qualitative analysis as shown in the PRISMA flow chart (Fig. 2).

### Characteristics of the included studies

**Fig. 3:**
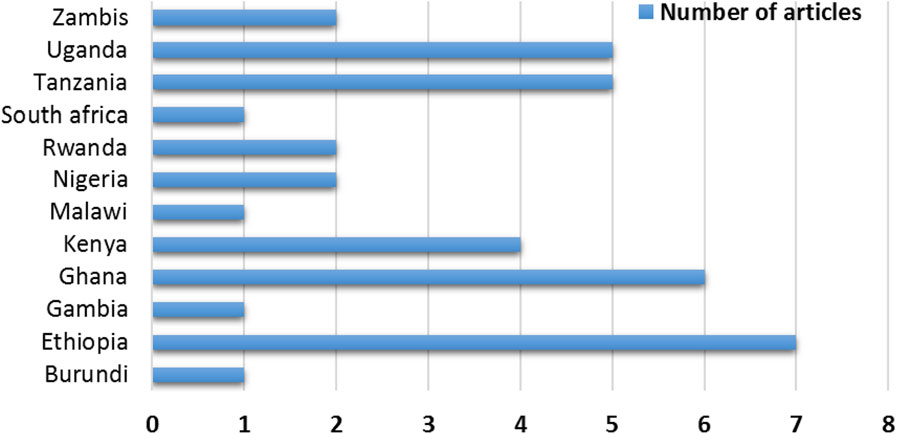
Distribution of the included articles by country in which they were conducted

For nearly a third of the included studies (35.13%), data collection took place in 2012. Twenty-seven percent of the articles were published in 2014 while 24% published in 2016. Fifteen (40.6%) of the included articles were conducted with qualitative study designs [23, 38, 43, 44, 46, 48, 54, 55, 63, 65, 66, 68, 69, 72, 73], while 17 (45.9%) were quantitative [39–42, 45, 47, 52, 53, 57–62, 64, 67, 70] and five (13.5%) were mixed-methods studies [49–51, 56, 71]. Almost all of the studies were facility-based cross-sectional surveys. The included articles were from 12 sub-Saharan African countries. The majority (70.3%) of the articles were from Uganda, Tanzania, Kenya, Ghana, and Ethiopia (Fig. 3). According to the MMAT quality assessment results, 31 articles met all of the quality criteria (100%) [38–40, 44, 46–49, 51–70, 72, 73], and 6 fulfilled 3 criteria (75%) [41–43, 45, 50, 71]; hence, all studies were included in the narrative synthesis.

### Barriers to seeking emergency obstetric care

The reported barriers to seeking healthcare were grouped using the three delay model and thematically presented in the following sections.

#### Delay I: Delay in decision to seek care

Five main sub-themes emerged within delay I, which included (i) socio-economic factors, (ii) community perceptions about obstetric complications, (iii) women’s autonomy and insufficient male partner involvement in the management of obstetric emergency, (iv) maternal obstetric history and health service utilization, and (v) women’s knowledge about obstetric danger signs. These factors resulted in delays at home to seeking appropriate obstetric care in a timely manner when needed.

i. Socio-economic factors

In this review, socio-economic factors were reported in several studies to hinder women from accessing and utilizing EmOC when they experienced obstetric complications. It was reported that younger women [53, 59] were more likely to delay in seeking obstetric care during an obstetric emergency [62]. Delays at home were more likely to be observed among women who could not read or write [49, 73], those who attended only primary education [53, 61, 62], and unemployed women [53, 61, 62]. Women who were unable to speak the language spoken by their service providers [49], those living in a rural area [40, 47, 49, 53, 64, 66, 67, 69], and those who experienced unintended pregnancy [53] were more likely to delay in seeking care when needed. Researchers indicated that women with a low household income were more likely to delay seeking healthcare, regardless of the need for EmOC services [39, 40, 49, 51, 59, 63].

ii. Community perceptions about obstetric complications

Findings of the included studies reported that many members of the community believed that obstetric complications were normal events that occur during pregnancy [50, 66] and others believed they were caused by witchcraft [53, 66]; therefore, they thought that seeking healthcare would be of no help. There was a reported preference to delay seeking help as people expected obstetric complications would resolve with no interventions [63], and many women preferred to consult traditional healers [39, 45, 73], which also resulted in delays in seeking medical services. In some studies, it was indicated that women preferred to visit traditional birth attendants (TBA) [58, 68] compared to modern medicine, which also resulted in delays in seeking EmOC services from a health facility.

iii. Women’s autonomy and insufficient male involvement

In this review, the findings of several studies showed that women usually lacked decision-making autonomy [46, 49] and they were required to obtain permission from family members, including their husband [45, 63, 73], in order to access health services when they experienced obstetric complications. In addition, it was reported that husbands were often absent during an obstetric emergency [43] although the majority of husbands provided financial and emotional support for their wives [43, 61, 62]. The main reasons reported for insufficient male physical involvement were lack of accommodation for men in the facilities [43] and the fact that in these cultures, a man is not allowed to witness the delivery of a baby [61].

iv. Maternal obstetric history and health service utilization

Researchers showed that women who have had many children [51] and experienced several pregnancies [59, 62, 73] were more likely to delay seeking EmOC services when they faced obstetric complications. It was indicated that women who experienced obstetric complications [46, 66] and those who visited health facilities for antenatal care (ANC) services during previous pregnancies [39, 45, 59, 61, 62] were less likely to delay seeking EmOC. It was also indicated that women with good birth preparedness and complication readiness (BPCR), which was defined as having plans for a birth attendant, birth location, arranged transport, identified a blood donor, and saved money in case of an obstetric complication [57], were less likely to delay in seeking EmOC during obstetric emergencies [39, 57].

v. Women’s knowledge of obstetric danger signs

In study findings, it was shown that delays in seeking EmOC during obstetric emergencies were more likely among women who lacked knowledge of obstetric danger signs [45, 59, 68]. Some women might perceive the danger signs to be normal, though they are knowledgeable about the danger signs, [66] and uncertain about the severity of the problems [73] that led them to delay seeking care. Delays in recognizing obstetric danger signs were found to be more likely among uneducated women [49, 53] and that women who attended some formal education [39] were more likely to recognize obstetric danger signs. Those women who lived in rural areas [28, 63, 66, 73] and who lacked information about the availability of EmOC services [49, 51] were also more likely to delay seeking obstetric care during an obstetric emergency.

#### Delay II: Delay in reaching a health facility

The second delays were reported in several studies, and three sub-themes emerged from this category of delays. The sub-themes included (i) availability of transportation infrastructure, (ii) distance from the health facility, and (iii) lack of finance for transportation. These barriers resulted in delays on the way to the health facility even if the decision to seek care was made in a timely manner.

i) Poor transport infrastructure

Lack of transportation infrastructure is reported in the majority of the studies in delaying women on the way to health facilities and was also related to a lack of vehicles for travel [23, 39, 45, 49, 51, 59, 65, 66, 68] and poorly designed road infrastructure in rural villages [52, 63, 66, 68, 72]. For example, Kakaire et al. (2011) reported that seasonal rain made some roads inaccessible even while it was available [63], which further delayed women in reaching a health facility. Timely access to appropriate EmOC services was also reported to be hampered by a shortage of ambulances in a number of studies [51, 56, 66, 69, 72]. Further, it was indicated that a shortage of ambulances deterred healthcare providers from referring clients, which resulted in delays in reaching referral EmOC facilities [41, 72].

ii) Distance from health facilities

Distance from a health facility and the considerable travel times to health facilities are influential factors found to affect women’s access to EmOC facilities [42, 46, 51–53, 60, 63, 64, 68]. Researchers reported that women were delayed in reaching a health facility because of the long distance required to travel [68, 72]. It also might be difficult for labouring women to travel long distances because they were not healthy enough. These constraints could be related to the health system perspective of poorly located obstetric centres [52] and insufficient numbers of EmOC facilities for the users within a recommended distance [55].

iii) Lack of finance for transportation

Lack of money to cover transportation fees to travel to a health facility was reported in several studies as a source of delays for women in accessing a health facility when they faced obstetric emergencies [42, 45, 46, 51, 58, 63, 66, 70, 72]. In developing countries, women usually lack money to cover transportation fees to travel to a health facility [30]. These challenges may delay or totally prevent mothers in need of emergency obstetric care from seeking and using the service when they are in need.

#### Delay III: Delay in receiving healthcare at the facility

A number of factors that resulted in the third level of delays were reported in different studies and three sub-themes emerged from this category. These sub-themes included (i) lack of EmOC services and supplies, (ii) shortage of trained human resources, and (iii) management of EmOC services. These challenges resulted in delays in receiving timely appropriate care after women reached a health facility.

i. Lack of EmOC services and medical supplies

Though the performance of the signal functions of EmOC facilitates utilization of the service, the majority of health facilities were reportedly unable to perform some signal functions of EmOC [41, 47, 51, 67]. In some countries, the availability of EmOC facilities was below the UN standard of five EmOC facilities per 500,000 population [48, 65, 67]. Many health facilities lacked necessary medical equipment [41, 49, 55, 60, 69, 70] and essential drugs [41, 48, 50, 55, 56, 60, 68, 69, 71], challenging provision of the services. As a result, provision of sub-standard care [39, 45, 58, 65, 68–72] was reported at several health facilities. A shortage of infrastructure including a shortage of beds [41, 46] and a lack of labour [49] and postnatal rooms [41] were also reported to delay the provision of EmOC services. In some studies, delays in the provision of services occurred due to irregular supply of electricity [50] and lack of an operation room [41] and an intensive care unit [45]. Lack of blood supply was reported several times as a factor in delaying the provision of lifesaving EmOC services [45, 63, 68]. In some studies, clients complained about the absence of toilets [38] and lack of water supply [46, 50] as a challenge to seeking care from EmOC facility.

ii. Healthcare providers’ training and attitude

In several studies, it was indicated that the majority of health facilities in sub-Saharan Africa were unable to provide some signal functions of EmOC due to a shortage of suitably trained personnel [38, 41, 44, 48–50, 54, 69, 71]. Because of this shortage of human resources, a longer waiting time to receive the service [41, 45, 52, 65, 66, 68] was reported. In the majority of instances, a lack of training was observed among care providers [41, 44, 48, 50, 51, 56, 60, 69–72], which was a risk factor for misdiagnosis and inappropriate treatment of patients [39, 45, 58, 69, 70, 72]. Poor provider-client relationship was reported in the form of poor attitude of the providers [51, 54, 60, 65], lack of emotional support [44], and inflicting physical abuse to patients [58] that affected care-seeking behaviour. In some studies, it was indicated that the providers did not revisit the clients for a long time [46], and they provided judgemental services [48] by excluding others for no reasons. In some instances, it was reported that healthcare providers did not ensure the privacy of clients [41, 44, 58, 60, 65] during service provision.

iii. Management of emergency obstetric care

Factors related to poor management of EmOC were reported in a number of articles to pose a challenge to providing appropriate EmOC service. Poor management of EmOC including a lack of supportive supervision [23, 48, 50, 54, 69], a delayed patient referral system [41, 42, 45, 68], and poor staff motivation [41, 51, 54, 55, 72] were frequently reported. Findings of several studies showed that there was heavy workload [48, 49, 54, 69] at health facilities due to high staff turnover [23, 69] that was a result of a poor management system. This was reported to lead to patient overcrowding at the health facility [23, 38, 41, 46]. Furthermore, researchers indicated that there was a poor communication system among providers [44, 60, 65, 72]. Lack of implementation guidelines and protocols [48, 50, 54, 71] and unaccountability among care providers [54, 55, 69] were also reported to hamper the provision of appropriate EmOC services.

## Discussion

The main themes and sub-themes presented in the current review showed that barriers occurred at different levels. Some of these barriers were within women’s control; others were slightly outside of their control and barriers at the facility level, which were completely out of women’s control. This review utilized the three delays model to illustrate how different societal and institutional factors resulted in delays to seek obstetric care when needed. Factors related to the first delays were thematically grouped into five sub-themes, and specific factors affecting women’s decision-making ability were detailed under each sub-theme. Similarly, three sub-themes emerged from the second and third delays, and specific barriers experienced by clients and the service providers’ perspectives were presented under each sub-theme.

In the majority of the articles, decision-making to seek obstetric care during obstetric emergencies was found to be impeded by different factors. As Thaddeus and Maine (1994) have pointed out, the actual decision-making process is determined by the reluctance of the patients to seek healthcare and perceived and actual barriers experienced by the clients [28]. Likewise, in our systematic review, socio-economic factors were found to play a crucial role in delaying decision-making to seek EmOC when needed. Most of the identified socio-economic barriers in the current review were consistent with other study findings in developing countries that delayed decision-making in seeking obstetric care was more likely to be observed among younger women [74–76] and uneducated mothers [20, 74, 76–78]. Delays in accessing obstetric care were frequently reported among women from a low household income [20, 29, 74, 75, 78–81] who lacked financial resources. Similarly, we identified that delays among unemployed women [20, 74, 80] who lived in rural areas [80, 82] with lower access to media [29, 74, 80, 81] were commonly reported.

Perceptions of women and the general community have been identified as an important influence on women’s decision to seek appropriate healthcare, especially during the perinatal period [50, 56]. Findings from a number of studies identified that women prioritize their privacy [41, 44, 65]; hence, they prefer to visit a traditional healer where privacy is relatively maximized. Women with obstetric complications may fear unwelcome procedures, such as intrusive vaginal exams and painful surgical interventions [38, 72], at a health facility. The findings of our review are in agreement with other findings of systematic reviews in developing countries [80, 82–84]. Similar study findings were reported from developing countries including Indonesia [85] and Bangladesh [75, 81] that the perception of the community has impacted the decision-making time for women to seek timely obstetric care when needed.

In the current review, a lack of decision-making autonomy among women and the influence of male partners were reported in several articles to affect the length of time to seek obstetric care. This is the main barrier for many women in developing countries, which affected women’s access to EmOC, as they have to wait for permission from their spouse and other family members [29, 78, 79, 82–84]. Researchers in other developing countries also supported the current finding that women who utilized ANC were less likely to delay in deciding to seek obstetric care when they experienced obstetric emergencies [74, 76, 84, 86]. This might be attributed to the counselling services women receive during ANC visits [87]. A delay in decision-making was also less likely among women who did not experience previous birth complications [74].

In the findings of several studies in our review, it was indicated that most women were unable to recognize the symptoms of pregnancy complications due to the lack of knowledge about obstetric danger signs. Delays in seeking care for obstetric complications were found to be more likely among women with a lower level of knowledge about obstetric danger signs. Several study findings from developing countries supported the current review in that women with a lower level of knowledge about obstetric danger signs were relatively more delayed in seeking obstetric care [75, 76, 80–82, 85]. In other studies, similar findings to ours have been reported; a lower level of women’s knowledge about obstetric danger signs was more likely among uneducated women [75, 81, 82].

This review revealed that the unavailability of transportation options played a central role in whether or not a facility could be reached in a timely manner. The majority of the second delays occurred due to a shortage of vehicles, poor road infrastructure, and shortage of ambulances. In the absence of a reliable transportation, women used difficult modes of transportation including bicycles, which take longer to reach a facility [84]. In some rural areas, local public transportation, which is the only means available, was often erratic, and the transportation fee was prohibitively expensive [29, 88]. Furthermore, it is usually difficult for women from a lower household income to afford transportation fees [29, 75, 77, 79, 81, 82]. Shortage of ambulances [29, 77, 79–84, 89, 90] for patients and distance from health facility [20, 29, 74, 77, 79–86, 89] were also found to be major barriers delaying women on the way to a health facility. Poor topographical access to a health facility was consistently reported to be an essential cause of the second delays [77, 79, 85].

Availability of EmOC services and necessary medical supplies eases the access to and utilization of the service for women when they face obstetric emergencies [41, 47, 67]. In the current review, a lack of EmOC service and a shortage of medical supplies were found to be the major causes of the third delays. Shortage of utilities including insufficient beds, shortage of rooms, and irregular supply of electricity and water supply were also reported to delay service provision. Likewise, the current findings were in line with studies in developing countries where barriers due to unavailability of service [20, 82, 90, 91] and a lack of essential drugs and medical supplies [82, 83, 90–92] resulted in delays in the health facility. In developing countries, a lack of an operation theatre [83, 90] and a shortage of maternity service rooms [80, 84, 92] consistently affected the provision of EmOC services in health facilities. Similar to the findings of the current review, in different developing countries, lack of blood supply [84, 90] and insufficient utilities [20, 82, 84, 90] was found to affect the length of time to provide EmOC services.

In this review, delayed EmOC service provision due to factors related to lack of human resources was reported in several articles. This impediment of service provision resulted from a shortage of human resources was reported in different perspectives including lack of skill, shortage of staff, and poor attitude among providers. The findings of the current systematic review could be justified with other study findings conducted in developing countries where a shortage of competent human resource [82, 83, 90, 92, 93] and lack of training [29, 80, 82, 90–92, 94] resulted in delays to provide quality obstetric care. Similarly, unwelcoming providers’ attitude [82–84, 91] and staff absenteeism [80, 82] were reported to be the primary cause of delays that occurred in health facilities.

Problems related to the management of EmOC services were reported in many articles to pose a challenge to the provision of effective EmOC services. Similar to the findings of the current review, in developing countries, an absence of supportive supervision [83, 94] and poor staff motivation mechanism [82, 90, 92, 93] impeded the provision of quality EmOC services. Poor liaison among health facilities affected patient referral [29, 80, 82, 89, 92, 94] and resulted in unnecessary delays. Evidence supporting the current review was reported in different studies. Poor communication among providers [77, 82, 90, 94], poor coordination of service provision [29, 82, 90], reluctance to implement guidelines [84, 91, 92, 94], and unaccountability among facility managers [29, 82, 94] were reported to affect EmOC service provision.

The current review has the following strengths. Current evidence on the barriers to access and utilization of emergency obstetric care was systematically synthesized from both sides: the service providers’ and users’ perspectives. Methodologically, we included articles conducted with quantitative, qualitative, and mixed-methods study designs, rather than a homogeneous source; hence, wider findings were represented. Barriers reported in the quantitative studies were supported with descriptive factors obtained from the qualitative studies, which strengthened the review. Likewise, studies with a mixed-method design provided more wide-ranging barriers to access and utilization of emergency obstetric care. Generally, barriers to access and utilization of EmOC were identified from papers with a diversity of study designs and are presented using the standardized analytical framework. Screening for articles, data extraction, and evidence synthesis were conducted by two reviewers in order to strengthen the reliability of the study outcomes and minimize the subjectivity of the data extraction, evidence synthesis, and interpretation. Similarly, we strictly adhered to the inclusion and exclusion criteria, and the quality of articles was appraised using a validated quality appraisal method.

Beside these strengths, the limitations of the review should be noted. In a relatively fewer number of studies, researchers reported barriers from the service providers’ perspective so that few health system-related barriers were reported. We included only articles published in English and indexed in MEDLINE, CINAHL, Embase, Psych Info, and Maternity and Infant Care databases. Gray literature, including government reports, was not included in the review; hence, there is a possibility that some potentially relevant studies indexed elsewhere or published in a language other than English may have been missed. Nevertheless, a comprehensive search for articles offered identification of major barriers to access and utilization of EmOC care in sub-Saharan Africa.

## Conclusion

Different factors were found to hamper access and utilization of EmOC among women in sub-Saharan Africa. Very complex and multi-faceted barriers delayed women’s access to and utilization of EmOC when they are in need. These barriers are inter-dependent and occur at multiple levels. These multiple barriers require multi-dimensional interventions that can take into consideration the challenges of the service providers’ and users’ perspectives. Though the factors are nearly similar across sub-Saharan African, country-specific interventions that are able to overcome the identified barriers are needed to tackle these challenges. Intersectoral collaboration should be strengthened, and all stakeholders should cooperate and work together to alleviate barriers that are observed at multiple levels. Health insurance should be made available for women to improve the effect of economic liability on health service utilization.

Investments in healthcare infrastructure, including roads facilities, obstetric care facilities, toilet facilities, water supply, interpersonal communication, equipment, human resources for health, and community-based health information dissemination, may lead to improved access to emergency obstetric services. Future research should focus on the exploitation of new opportunities that are helpful to eliminate these barriers and improve women’s access to and utilization of EmOC. Further research should focus on the third delays, as little is known about facility readiness to render quality EmOC.

## Additional files

Additional file 1: The MEDLINE sample search strategy. (DOCX 14 kb)

Additional file 2: Result of quality appraisal, the Mixed Methods Appraisal Tool (MMAT). (DOCX 18 kb)

Additional file 3: Characteristics of the included studies. (DOCX 24 kb)

## Abbreviations

ANC: Antenatal care
AVD: Assisted vaginal delivery
BEmOC: Basic emergency obstetric care
BPCR: Birth preparedness and complication readiness
CEmOC: Comprehensive emergency obstetric care
CINAHL: Cumulative Index to Nursing and Allied Health Literature
EmOC: Emergency obstetric care
HCW: Healthcare workers
MDGs: Millennium development goals
MMAT: Mixed Method Appraisal Tool
MMR: Maternal mortality rate
PICOS: Participant interventions comparison outcome setting
PRISMA: Preferred Reporting Items for Systematic Reviews and Meta-Analyses
SDGs: Sustainable development goals
UNs: United Nations
WHO: World Health Organization
