# Supplementary material for: Barriers to access and utilization of emergency obstetric care at health facilities in sub-Saharan Africa: a systematic review of literature
Source: Syst Rev. 2018 Nov 13;7:183. doi: 10.1186/s13643-018-0842-2 (PMC6234634; doi:10.1186/s13643-018-0842-2)
Supplement: Supplementary file 1 — The MEDLINE sample search strategy. (DOCX 14 kb) [file 13643_2018_842_MOESM1_ESM.docx]

Database(s): **Ovid MEDLINE(R) Epub Ahead of Print, In-Process & Other Non-Indexed Citations, Ovid MEDLINE(R) Daily and Ovid MEDLINE(R)**1946 to Present 
Search Strategy:

| **#** | **Searches** | **Results** |
| --- | --- | --- |
| 1 | Pregnancy Complications/ or "emergency obstetric care".mp. or Obstetric Labor Complications/ | 100298 |
| 2 | "emergency obstetric and newborn care".mp. or Delivery, Obstetric/ or Emergency Medical Services/ | 64973 |
| 3 | EmOC.mp. | 233 |
| 4 | EmONC.mp. | 76 |
| 5 | Postpartum Hemorrhage/ or maternal h?emorrhage.mp. or Placenta Previa/ | 9202 |
| 6 | Maternal infection*.mp. | 1703 |
| 7 | prolonged labo?r.mp. or Dystocia/ | 4384 |
| 8 | Abortion, Induced/ or "complication* of abortion".mp. or Abortion, Spontaneous/ | 42030 |
| 9 | hypertension during pregnancy.mp. or Pre-Eclampsia/ or Hypertension, Pregnancy-Induced/ | 30513 |
| 10 | Cesarean Section/ or Caesarean.mp. | 50528 |
| 11 | "blood transfusion".mp. or Blood Transfusion/ | 83380 |
| 12 | Vacuum Extraction, Obstetrical/ or Vacuum extraction.mp. | 1900 |
| 13 | Oxytocin.mp. or Oxytocin/ | 26047 |
| 14 | Parentral administration of Magnesium Sulfate/ or Magnesium sulphate.mp. | 1472 |
| 15 | Oxytocin/ or Intravenous administration of oxytocin.mp. | 18820 |
| 16 | intravenous administration of antibiotic.mp. | 7 |
| 17 | Placenta, Retained/ or Manual removal of placenta.mp. | 728 |
| 18 | assisted vaginal delivery.mp. | 318 |
| 19 | 1 or 2 or 3 or 4 or 5 or 6 or 7 or 8 or 9 or 10 or 11 or 12 or 13 or 14 or 15 or 16 or 17 or 18 | 373217 |
| 20 | (barrier* or obstacle* or factor* or Challenge* or determinant* or access* or utiliz* or Utilis* or hinder* or hindrance* or impede* or impediment*).mp. | 6499762 |
| 21 | sub-Saharan Africa.mp. or exp "Africa South of the Sahara"/ | 189104 |
| 22 | 19 and 20 and 21 | 3868 |
| 23 | animals/ not (humans/ and animals/) | 4496223 |
| 24 | 22 not 23 | 3858 |
| 25 | limit 24 to (english language and yr="2010 -Current") | 1771 |
