# Supplementary material for: Barriers to access and utilization of emergency obstetric care at health facilities in sub-Saharan Africa: a systematic review of literature
Source: Syst Rev. 2018 Nov 13;7:183. doi: 10.1186/s13643-018-0842-2 (PMC6234634; doi:10.1186/s13643-018-0842-2)
Supplement: Supplementary file 2 — Result of quality appraisal, the Mixed Methods Appraisal Tool (MMAT). (DOCX 18 kb) [file 13643_2018_842_MOESM2_ESM.docx]

**Result of quality appraisal, the Mixed Methods Appraisal Tool (MMAT)**

1. **Qualitative study**

| Qualitative studies | Quality assessment components using MMAT | | | | |
| --- | --- | --- | --- | --- | --- |
|  | Are the sources of data relevant to research question? | Is the data analysing process relevant to address research question? | Is appropriate consideration given to how findings relate to context? | Is appropriate consideration given to how findings relate to researchers’ influence? (Reflexivity) | Total |
| Wright K et al (2017) | * | * | * | * | 4* |
| Echoka E et al (2014) | * | * | * | * | 4* |
| Austin A et al (2015) | * | * | * | * | 4* |
| Story T W et al (2016) | * | * | * | 0 | 3* |
| Stal KB et al (2015) | * | * | * | * | 4* |
| Sialubanje S et al (2015) | * | * | * | * | 4* |
| Paul M et al (2014) | * | * | * | * | 4* |
| Mkoka DA et al (2014) | * | * | * | * | 4* |
| Mkoka DA et al (2014) | * | * | * | * | 4* |
| Jammeh A et al (2011) | * | * | * | * | 4* |
| Ganle JK et al (2014) | * | * | * | * | 4* |
| Thorsen VC. et al. (2012) | * | * | * | * | 4* |
| Chi PC et al (2015) | * | * | * | * | 4* |
| Afari H et al (2014) | * | * | * | * | 4* |
| Aborigo RA et al (2014) | * | * | * | * | 4* |
| Total=15 |  |  |  |  |  |

1. **Quantitative non-randomized studies**

| Quantitative non-randomized studies | Quality assessment components using MMAT | | | | |
| --- | --- | --- | --- | --- | --- |
|  | Are participants recruited in a way that minimizes selection bias? | Are measurements appropriate  regarding the exposure or intervention and outcomes? | In the groups being compared, are the participants  comparable, or do researchers take into account? | Are there complete outcome data (80% or above), an acceptable response rate (60% or above), or an acceptable  follow-up rate for cohort studies? | Total |
| Soma-Pillay p et al (2016) |  | * | * | * | 3* |
| Tayler-Smith K et al (2013) | * | * |  | * | 3* |
| Ueno E et al (2015) | * | * |  | * | 3* |
| Phiri SN et al (2016) | * | * | * | * | 4* |
| Mutua MM et al (2015) | * | * | * | * | 4* |
| Mbalinda SM et al (2014) | * | * | * | * | 4* |
| Liambila WL et al (2014) | * | * | * | * | 4* |
| Lakew S et al (2015) | * | * | * | * | 4* |
| Kumsa A eta al (2016) | * | * | * | * | 4* |
| Kakaire O et al (2011) | * | * | * | * | 4* |
| Gudu W (2017) | * | * | * | * | 4* |
| Worku AG et al (2013) | * | * | * | * | 4* |
| Carnahan LR et al (2016) | * | * | * | * | 4* |
| Wilunda c et al (2013) | * | * | * | * | 4* |
| Niyitegeka J. et al. (2017) | * | * | * | * | 4* |
| Kalisa R. et al. (2016) | * | * | * | * | 4* |
| Echoka E. et al. (2013) | * | * | * | * | 4* |
| Total = 17 |  |  |  |  |  |

1. **Mixed Methods studies**

| Qualitative studies | Quality assessment components using MMAT | | | | |
| --- | --- | --- | --- | --- | --- |
|  | Are the sources of data relevant to research question? | Is the data analysing process relevant to address research question? | Is appropriate consideration given to how findings relate to context? | Is appropriate consideration given to how findings relate to researchers’ influence? (Reflexivity) | Total |
| Oguntunde O et al (2015) | * | * | * | * | 4* |
| Nwameme AU et al (2013) | * | * | * | * | 4* |
| Braddick L et al (2016) | * | * | * | * | 4* |
| Oiyemhonlan B et al (2013) | * | * | * | * | 4* |
| Mirkuzie AH et al (2014) | * | * | * | * | 4* |
| Total = 5 |  |  |  |  |  |

| Qualitative descriptive component | Quality assessment components using MMAT | | | | |
| --- | --- | --- | --- | --- | --- |
|  | Is the sampling strategy relevant to address the quantitative research question? | Is the sample representative of the population under study? | Are measurements appropriate (clear origin, or validity known, or standard instrument)? | Is there an acceptable response rate (60% or above)? | Total |
| Oguntunde O et al (2015) | * | * | * | * | 4* |
| Nwameme AU et al (2013) | * | * | * | * | 4* |
| Braddick L et al (2016) | * | * | * | * | 4* |
| Oiyemhonlan B et al (2013) | * | * | * | * | 4* |
| Mirkuzie AH et al (2014) | * | * | * | * | 4* |
| Total = 5 |  |  |  |  |  |

| Mixed method design | Quality assessment components using MMAT | | |  |
| --- | --- | --- | --- | --- |
|  | Is the mixed methods research design relevant to address the qualitative and quantitative research  questions (or objectives), or the qualitative and quantitative aspects of the mixed methods question (or  objective)? | Is the integration of qualitative and quantitative data (or results) relevant to address the research  question (objective)? | Is appropriate consideration given to the limitations associated with this integration, e.g., the  divergence of qualitative and quantitative data (or results)? | Total |
| Oguntunde O et al (2015) | * | * | 0 | 2* |
| Nwameme AU et al (2013) | * | * | * | 3* |
| Braddick L et al (2016) | * | * | 0 | 2* |
| Oiyemhonlan B et al (2013) | * | * | * | 3* |
| Mirkuzie AH et al (2014) | * | * | * | 3* |
| Total = 5 |  |  |  |  |
