# Supplementary material for: Barriers to access and utilization of emergency obstetric care at health facilities in sub-Saharan Africa: a systematic review of literature
Source: Syst Rev. 2018 Nov 13;7:183. doi: 10.1186/s13643-018-0842-2 (PMC6234634; doi:10.1186/s13643-018-0842-2)
Supplement: Supplementary file 3 — Characteristics of the included studies. (DOCX 24 kb) [file 13643_2018_842_MOESM3_ESM.docx]

**Table S1: Characteristics of included studies**

| No | Author/year | Country  type | Study Design and data collection year | study Participants | **Study objectives** | **Outcomes of interest to the review** | **Quality assessment score (%)** |
| --- | --- | --- | --- | --- | --- | --- | --- |
| 1 | Soma-Pillay p. et al. (2016) | South Africa  EmOC | Quantitative  2013-2015 | 100 Mothers with any one of near-misses | To determine the delays/barriers in providing obstetric care to women who classified as a maternal near-miss | **1st delay:** Lack of knowledge of the problem, Inadequate ANC, Non-compliance with HCP advice, Belief in alternative care, Family member prevented woman from accessing healthcare **2nd delay:** Lack of finance, Lack of transport. **3rd delay:** Lack of identification of the problem, Delay in patient admission, referral or treatment, Lack of resources (blood/intensive care), Substandard care (inappropriate diagnosis or treatment) | 75 |
| 2 | Wright K. et al. (2017) | Nigeria  EmOC | Qualitative  2015 | 39 Women who received any of the eight signal function of EmOC | To explore the opinions of women regarding EmOC provided in public health facilities in Lagos | Inadequate number of staff, higher patient load, nurses lack respect for clients, dirtiness of toilet, lack of mosquito net for baby, Stress associated with blood donation, high treatment costs, | 100 |
| 3 | Echoka E. et al. (2014) | Kenya  EmOC | Qualitative  2010 | 30 Women who experienced maternal near miss | To explore barriers to emergency obstetric care by women who experienced life threatening obstetric complications in Malindi District, Kenya | **Delay in decision**: Knowledge of danger signs, perceived the danger signs to be normal during pregnancy, Perceived severity of the illness, previous uncomplicated pregnancies, lack of money. **Delay in reaching facility**: Living in rural areas, t sought care firs from dispensary or health centre, lack of ambulance, poor road infrastructure and lack of transportation. **Delays in receiving** treatment: long waiting time, unavailability of HCW | 100 |
| 4 | Austin A. et al. (2015) | Ethiopia  EmOC | Mixed method  2013 | 29 Medical director, liaison officer, MCH coordinator and senior nurse (qual)  111 maternal health services Providers (quant) | To assess barriers to the provision of emergency obstetric care from the perspective of healthcare providers in Addis Ababa, Ethiopia | lack of transportation and Pre-referral communication gaps, overcrowding at the referral hospital, insufficient pre-service and in-service training, and absence of supportive supervision, staff turnover/rotation, No formal guidelines or protocol for supportive supervision, Unscheduled, random supervisory visits, Punitive and document-based supervisory models, non-standardized donor-driven specific training | 100 |
| 5 | Ueno E. et al. (2015) | Tanzania  EmOC | Quantitative  2012 | 204 HCPs working at maternity and RCH units | To describe cadres of HCPs who are considered SBAs in Tanzania, the EmOC signal functions they perform and challenges associated with performance of EmOC signal functions | Non-availability if signal functions, lack of equipment and supplies, lack of human resource, lack of knowledge and skills to perform EmOC, lack of training, patient load, shortage of bed, shortage of rooms, lack of stock of blood, lack of drugs/medicine, lack of operating room, lack of privacy, lack of working space, shortage of ambulance, poor transportation, late referral, poor staff benefit, lack of team work and cooperation. |  |
| 6 | Tayler-Smith k. et al. (2013) | Burundi  EmOC | Quantitative  2011 | 1478 Ambulance call by women with obstetric complication | To cost of ambulance service and association between referral times and maternal and early neonatal deaths and impact of the referral service on coverage of complicated obstetric | Transport cost, lack of communication, distance from facility, delay in referral | 75 |
| 7 | Story WT. et al. (2016) | Ghana  EmOC | Qualitative  2012 | 39 (21 mothers and 18 fathers) who experienced obstetric emergencies | To assess Male Involvement and Accommodation During Obstetric Emergencies in Rural Ghana | Partner non-involvement to provide financial or emotional support in emergency care, male unaccompany their wifes, facilities had not accommodated the male partner | 75 |
| 8 | Stal KB. et al. (2015) | Tanzania  EmOC | Qualitative  2011-2012 | 19 mothers and 3 health workers | To assess perceptions of the quality of obstetric care of women who delivered in a rural Tanzanian referral hospital. | lack of trust TBA’s ability to handle complications correctly, Lack of emotional support and rudeness among health workers, health professional negligence, lack of communication about medical conditions for women, Relatives were not allowed to enter the labour ward, shortage of labour ward and less beds, shortage of staff, lack of privacy, lack of communication with the patient between midwife and patient, lack of training, shortage of infrastructure (rooms, beds) to meet the increased patient need. | 100 |
| 9 | Sialubanje C. et al. (2015) | Zambia  EmOC | Qualitative  2014 | 32 Women of reproductive age group | To assess access to skilled facility-based delivery services: Women’s beliefs on  facilitators and barriers to the utilisation of maternity waiting homes in rural Zambia | Decisions being made by husband, having no previous complications, distance from facility, luck of fund, being occupied with harvest (other work), lack of beds or mattresses, overcrowded space, lack of food for pregnant women during stay, lack of water at the facility, sanitation was poor, nurses or midwives not checking mothers for long time | 100 |
| 10 | Phiri SN. et al. (2016) | Zambia  EmOC | Quantitative  2010 | 2114 women in urban and 1226 in rural Record of women who gave birth in EmOC facility | To to assess the deficit in life-saving obstetric services in the rural and urban areas of Kapiri Mposhi district | Rural urban inequality, Lack of equipment, policy recommended referral of women with sepsis without giving antibiotic, policy reccomand referral for prolonged labor without attempting AVD, non-availability of service (CS), non-availability of EmOC | 100 |
| 11 | Paul M et al. (2014) | Uganda  Abortion | Qualitative  2012 | 27 health care providers | To explore physicians’ and midwives’ perception of post-abortion care with regard to professional competences, methods, contraceptive counselling and task shifting/sharing in post-abortion care | Norms and values, abortion was perceived as immoral and as murder by the users, social stigma, legal restrict, Health care providers’ bad attitude, lack of in-service training, absence of service that are provided together (FP), lack of equipment, lack of knowledge among providers, no hospital guidelines  and a lack of available drugs in the health facilities, shortage of equipment, an absence of doctors and a lack of skills. poor supervision, and too  heavy a workload, lack of task shifting | 100 |
| 12 | Oiyemhonlan B. et al. (2013) | Ghana  EmOC | Mixed  2012 | 2 emergency obstetric cases, 29 antenatal focus group discussants and 5 midwives at the maternity unit | To to identify obstetric emergencies and barriers to emergency care seeking; examine the perspective of midwives regarding their role in maternity care and management of obstetric emergencies, and explore women’s knowledge and response to obstetric | Lack of knowledge of obstetric emergency, poor health seeking behaviour for obstetric complications, lack of information about service, lack of transportation, language barriers when communicating with patients, lack of essential resources to perform tasks, and a lack of physical space in the maternity ward, shortage of human resource, shortage of bed, stress of HCP. Individual level: high rates of illiteracy, poor interpretation and recognition of obstetric emergencies, poverty and lack of personal or arranged transportation to the hospital, as well as language and cultural barriers. Community level: Lack of hospitals and healthcare providers in rural areas, a lack of easily accessible transportation and tangentially a lack of women empowerment. system level: shortage of staff and resources | 100 |
| 13 | Oguntunde O. et al. (2015) | Nigeria  HTN | Mixed  2012 | 30 health managers and 80 healthcare providers form 80 health facilities | To examined facilitators and barriers to the use of magnesium sulphate (MgSO4) in the management of pre-eclampsia/ eclampsia (PE/E) in health facilities in Bauchi and Sokoto States in Nigeria. | Non-availability of drug, drug stock out, non-availability of guideline and protocol, lack of supportive supervision, Inadequate staffing, inadequate training, lack of equipment, lack of electricity and water in the room, community perceive convulsion as evil sprit and not coming to facility earlier. | 75 |
| 14 | Nwameme AU. et al. (2014) | Ghana  EmOC | Mixed  2012 | 390 ANC attendees and health care workers | To assess compliance with Emergency Obstetric Care Referrals Among Pregnant Women in an Urban Informal Settlement of Accra, Ghana | Lack of knowledge of availability of service, lack of transportation, lack of ambulance, non accompany by staff during referral, distance from facility, lack of money, having good trust on the locally available health service, having more number of children, Financial problems, poor attitude of nurses at the referral centres, fear of surgery, Ignorance, previouse bad experience at the facility, non availability of service, lack of training, lack of support and feedback, lack of bed, doctors not always available at facility. |  |
| 15 | Mutua MM. et al. (2015) | Kenya  Abortion | Quantitative  2012 | 350 facilities | To assess factors that are associated with delays in seeking post-abortion care among women in Kenya | Young women aged less than 20 years, uneducated, married, unwanted pregnancies and those referred from other facilities had longer delays before seeking care, confessing Muslim faith, rural residence, unemployed/housewife, severity of problem, contraception use |  |
| 16 | Mkoka DA. et al. (2014) | Tanzania  EmOC | Qualitative  2012 | 16 facility managers | To explore the experiences of a district health management team in implementing Emergency Obstetric Care (EmOC) related policies and identifying emerging governance aspects. | Most challenges faced during the implementation of EmOC were related to governance issues at different levels and included delays in disbursement of funds from the central  government, shortages of health workers, unclear mechanisms for accountability, lack of incentives to motivate overburdened staffs and lack of guidelines for partnership development |  |
| 17 | Mkoka DA. et al. (2014) | Tanzania  EmOC | Qualitative  2012 | 17 facility managers | To describe the experience of rural health facility managers in ensuring the timely availability of drugs and medical supplies for emergency obstetric care (EmOC). | shortages of some essential drugs and supplies needed for EmOC, At management level: Long delay in supply of drug and medical supplies, Supply of expired and unmatched drugs and medical supplies, Delay in delivering care to women du to lack of material, Decreased trust in health workers, Decreased morale of health workers, Insufficient budget to meet local drug demand, lack of accountability and beaurocratic accessing fund and drugs from central management system | 100 |
| 18 | Mirkuzie AH. et al. (2014) | Ethiopia  EmOC | Qualitative  2013 | 24 Healthcare workers | To examine progress in the implementation of the BEmONC (BEmONC) in Addis Ababa. | insufficient knowledge and poor skill of professional, shortage of ambulance services, shortage of drug and supplies, lack of training, shortage of HCP, |  |
| 19 | Mbalinda SN. et al. (2014) | Uganda  EmOC | Quantitative  2012 | 810 women admitted in the antepartum | To assess the association between knowledge of danger signs and birth preparedness among women admitted with pregnancy complications | Poor knowledge of obstetric danger signs, poor birth preparedness and complication readiness | 100 |
| 20 | Liambila WN. et al. (2014) | Kenya  EmOC | Quantitative  2013 | 294 women aged 15–49 years | To assess the nature of childbirth related complications among the skilled and the non-skilled birth attendants in Western Kenya. | Undignified care, high delivery and transport costs and fear of hospital procedures such as HIV tests and mishandling of the placenta, disrespect and abuse during labour, physical abuse, non concented care, non confidencial care, discrimination, abandonment of care, considering the labouring women is strong, prefenrence for TBAs, poor quality of care at facility, high delivery cost, poverty, ignorance | 100 |
| 21 | Lakew S. et al. (2015) | Ethiopia  EmOC | Quantitative  2014 | 798 women who gave birth within one year | To assess women’s skilled assistance seeking behaviour for pregnancy complications among those who gave birth. | Unable to understand the seriousness of the complications, thought as unnecessary, and family disapproval, monthly household income, ANC use, age, Availability of transport access, Women education, Knowledge complications, birth order | 100 |
| 22 | Kumsa A. et al. (2016) | Ethiopia  EmOC | Quantitative  2014 | 304 women who gave birth in the last 12 months | To assess satisfaction with Emergency Obstetric and new born Care services  among clients using public health facilities in Jimma zone, Southwest Ethiopia | Shortage of staff. Lack of drug and equipment, transportation, availability of utilities, distance from facility, shortage of rooms, lack of communication, shortage of skill, simplicity of obtaining drugs, cost free drugs, poor attitude of providers, privacy, age of clients |  |
| 23 | Kakaire O. et al. (2011) | Uganda  EmOC | Quantitative  2010 | 140 women admitted as emergency obstetric referrals | To assess factors associated with birth preparedness, complication readiness and male participation in the birth plan among emergency obstetric referrals in rural Uganda | Husband involvement, Autonomy of the female, Age, age of spouse, parity, education level, occupation, occupation of spouse, education of spouse, ANC use |  |
| 24 | Jammeh A. et al. (2011) | Gambia  EmOC | Qualitative  2010 | 20 women who sought obstetric care and delivered in hospital | To assess explored barriers of timely access to emergency obstetric care services resulting in perinatal deaths and in survivors of severe obstetric complications in rural Gambia | Poor knowledge of obstetric danger signs, expecting improvement overtime, Lack of autonomy, lack of decision to seek care outside the home, Socio-Cultural, Traditional belief, Transportation, Distance, and Road Infrastructure, lack of many and service cost, lack of blood for transfusion, | 100 |
| 25 | Gudu W. (2017) | Ethiopia  HTN | Qualitative  2014/15 | 93 eclamptic women admitted to a general hospital | To assess health care seeking of patients in response to prodromal symptoms and timeliness of presentation after developing eclamptic and associated factors . | rural residence, distance from health facility of > 5km, illiteracy, | 100 |
| 26 | Ganle W. (2017) | Ghana  Barriers | Qualitative  2011/12 | 185mothers/20 HCP | To explore health system factors that inhibit women’s access to and use of skilled maternal and newborn healthcare services in Ghana despite these services being provided free. | limited and unequal distribution of skilled maternity care services, women’s experiences of intimidation in healthcare facilities, unfriendly healthcare providers, cultural insensitivity, long waiting time, limited birthing choices, poor care quality, lack of privacy at healthcare facilities, and difficulties relating to arranging suitable of transportation, poor communication, poor mutual trust and respect between caregivers and women, non-availability of service, lack of human resource | 100 |
| 27 | Worku AG et al. (2013) | Ethiopia  EmOC | Qualitative  2012 | 1,668 women who gave birth within one year | To assess experiences related to obstetric complication and seeking assistance from a skilled provider among women who gave birth in the last 12 months preceding the study. | Inability to judge the severity of morbidities, distance/transport problems, lack of money/ cost considerations and use of traditional options at home, Belonging to a wealthier quintile, getting antenatal care from a skilled provider and agreement of a woman in planning for possible complications, poor quality of service, use of home remedy options, lack of transportation, | 100 |
| 28 | Thorsen VC. et al. (2012) | Malawi  Barriers | Qualitative  2012 | 32 maternal death cases | To identify the socio-cultural and facility-based factors that contributed to maternal  deaths in the district of Lilongwe, Malawi. | Lack of recognizing signs, symptoms, and severity of the situation; using traditional Birth Attendant services; low female literacy level; delayed access to transport; hardship of long distance and physical terrain; delayed prompt quality emergency obstetric care; and delayed care while at the hospital due to patient refusal or concealment delay receiving treatment upon reaching the facility due to referral delays, missed diagnoses, lack of blood, lack of drugs, or inadequate care, and severe mismanagement. | 100 |
| 29 | Chi PC. et al. (2015) | Burundi  Uganda  EmOC | Qualitative  2013 | 32 local health providers and 37 staff of NGOs working in the area of maternal health | To explore the barriers to effective delivery of EmONC services in post-conflict Burundi and Northern Uganda | Unavailability of service, poor quality and distribution of EmONC services, shortage of trained qualified staff; lack of essential installations, supplies and medications; increasing workload, burn-out and turnover; and poor data collection and monitoring systems, demoralised personnel, lack of recognition and remuneration and lack of recognition; poor referral system; inefficient drug supply system; staff absenteeism in rural areas; and poor coordination among key personnel, weak curriculum; poor harmonisation and coordination of training; and inefficient allocation of resources, poor ambulance system, urban/rural inequity disterbuting EmOC resources | 100 |
| 30 | Carnahan LR. et al. (2016) | Tanzania  PPH | Quantitative  2015 | 115 healthcare providers at maternity service | To investigate healthcare providers’ knowledge and practices associated with prevention and management of postpartum hemorrhage to reduce maternal morbidity and mortality | Lack of knoeledge of HCP to diagnosis PPH, lack of misoprostol supply, length of job experience, transportation and financial constraints, unavailability of good services  at the health center, inadequate drug supplies, lack of Training | 100 |
| 31 | Braddick L. et al. (2016) | Uganda  PPH | Mixed  2014 | 154 maternal healthcare practitioners | To determine the level of adherence to postpartum hemorrhage clinical guideline and to explore context-specific barriers and facilitators to evidence-based obstetric care | Health system issue: Unreliable provision of medical supplies, Inadequate drug storage facilities, Staff shortages, Inadequate facilities. Knowledge issu: Lack of guideline awareness, Poor access to guidelines, Prioritizing experience over evidence, Incorrect clinical practice. Issue of updating skill: Lack of access to external training programs, Poor computer literacy and internet access, Prefer passive learning | 100 |
| 32 | Wilunda C. et al. (2013) | Ethiopia  EmOC | Quantitative  2010 | 760 women utilizing EmOC at Hospital | To measure equity in utilization of EmOC at Wolisso Hospital, Ethiopia | **Wealthier, urban, transportation** | 100 |
| 33 | Afari H. et al. (2014) | Ghana  EmOC | Qualitative  2012 | 18 HCWs | To describe system-based bottlenecks in designing strategies to improve referral processes to emergency obstetric care in rural Ghana. | Poorly Accessed road, inadequate transportation, High cost of transportation, lack of ambulance, Communication barriers, Clinical skill limitations, Unreliable standards of care and monitoring, prohibitive financial costs, sociocultural beliefs related to fear of blood transfusions and of death in bigger facilities, past negative experiences such as delays at hospitals, or inconsiderate treatment by hospital staff, poor recognising danger signs, lack of alerting receiving units, accompanying critically ill patients, documenting referral cases and giving and obtaining feedback on referred cases. | 100 |
| 34 | Aborigo RA. et al. (2014) | Ghana  EmOC | Qualitative  2010 | 35 women with newborn infants, 8 traditional birth attendants and local healers, 16 community leaders 13 health workers. 18 focus group discussions | To explored knowledge of obstetric danger signs among community members and the perceived factors that affect health seeking behaviour in rural northern Ghana | women’s uncertainty about the severity of symptoms or poor understanding of health messages, First-time mothers and illiterate women, families that prohibit hospital attendance or the use of allopathic medicine, using traditional healers, lack of autonomy, husband (other family member) take decision making role, lack of knowledge of danger signs, inability to early recognize symptoms | 100 |
| 35 | Niyitegeka J. et al. (2017) | Rwanda  EmOC | Quantitative  2015 | 441 mothers who underwent emergency cesarean | To describe the delays a mother faces when needing emergency cesarean delivery and its effect on neonatal outcomes in Rwanda | Delay in duration of labor prior to hospital admission, travel time, time from admission to surgical incision, and time from decision for emergency cesarean section to surgical incision, poor road infrastructure, lack of transportation and anbulance | 100 |
| 36 | Kalisa R. et al. (2016) | Rwanda  EmOC | Quantitative  2015 | 350 pregnant women who were admitted as referrals hospital | To assess level of male involvement in the birth plan and factors associated among obstetric referrals in rural Rwanda | Low male involvement, opposition of male accompany by HCPs, maternal education, occupation of spouce, personnel checked during ANC being community health worker | 100 |
| 37 | Echoka E. et al. (2013) | Kenya  EmOC | Quantitative  2010 | 40 Health facilities | to assess the actual existence and functionality of EmOC services at district level | none availability of EmOC services, Rural–urban inequities in geographical distribution of facilities, not providing sufficient life-saving | 100 |
